# Supplementary material for: Aberrant NSUN1 activity connects m5C-RNA modification to TDP-43 neurotoxicity in ALS/FTD
Source: Life Sci Alliance. 2025 Nov 4;9(1):e202503297. doi: 10.26508/lsa.202503297 (PMC12588883; doi:10.26508/lsa.202503297)
Supplement: Supplementary file 3 [file LSA-2025-03297_TableS2.docx]

**Aberrant NSUN1 Activity Connects m5C RNA Modification to TDP-43 Neurotoxicity in ALS/FTD**

Melissa Parra Torres^1^, Kumara Dissanayake^1^, James Gray^1^, Alistair J. Langlands^2^, Ridvan Kucuk^1^, Marek Gierlinski^3^, Claire Troakes^4,5^, Andrew King^5^, and Leeanne McGurk^1*^

^1^Molecular, Cell and Developmental Biology, School of Life Sciences, University of Dundee, Dow Street, Dundee DD1 5EH, UK

### ^2^National Phenotypic Screening Centre, School of Life Sciences, University of Dundee, Dow Street, Dundee DD1 5EH, UK

^3^Data Analysis Group, Division of Computational Biology, School of Life Sciences, University of Dundee, Dundee, DD1 5EH, UK

^4^Department of Basic and Clinical Neuroscience, Wohl Clinical Neuroscience Institute, Institute of Psychiatry, Psychology and Neuroscience, King’s College London, London SE5 9RX, UK

^5^London Neurodegenerative Diseases Brain Bank, SGDP Centre, PO65, Institute of Psychiatry, Psychology and Neuroscience, King’s College London, London SE5 8AF, UK

# ^6^King's College Hospital NHS Foundation Trust, Academic Neuroscience Centre

* To whom correspondence should be addressed: Dr Leeanne McGurk, Cell and Developmental Biology, School of Life Sciences, University of Dundee, Dow Street, Dundee DD1 5EH, UK. Email: [LMcgurk001@dundee.ac.uk](mailto:LMcgurk001@dundee.ac.uk)

**Table S2**: Details of patients analysed by immunohistochemistry or western blotting.

| **Case #** | **sex** | **Age at death** | **pm delay (hr)** | **clinical diagnosis** | **mutation status** | **application** |
| --- | --- | --- | --- | --- | --- | --- |
| 2 | M | 61 | 20.5 | MND (bulbar onset) | / | IHC |
| 3 | F | 63 | 25 | MND (limb onset) | / | IHC |
| 4 | F | 65 | 68 | MND | / | IHC |
| 5 | F | 68 | 51.5 | MND | / | IHC |
| 6 | M | 69 | 52.5 | MND | / | IHC |
| 7 | M | 71 | 58 | MND Motor Neurone Disease (limb onset) | / | IHC |
| 8 | M | 75 | 6.5 | MND | / | IHC |
| 9 | M | 75 | 35 | MND (limb onset) | / | IHC |
| 10 | F | 80 | 36.5 | Motor Neuron Disease (bulbar onset) | / | IHC |
| 11 | F | 90 | 34 | MND (bulbar onset) | / | IHC |
| 12 | F | 72 | 50 | Frontotemporal lobar degeneration with TDP-43 inclusions (FTLD-TDP ) (type C) | / | IHC |
| 13 | M | 73 | 29 | Frontotemporal Lobar degeneration-TDP-43 (FTLD-TDP type c) |  | WB |
| 14 | M | 73 | 58 | Frontotemporal lobar degeneration due to TDP-43 proteinopathy (type A) | / | WB |
| 15 | F | 67 | 27 | Frontotemporal lobar degeneration due to C9orf72 mutation | c9 | WB |
| 16 | F | 71 | 34 | Frontotemporal lobar degeneration due to TDP-43 proteinopathy (Type A) Alzheimer's disease BNE (Braak) stage IV | / | WB |
| 17 | M | 63 | 23 | Control |  | WB |
| 18 | F | 72 | 47 | Control | n/a | IHC |
| 19 | M | 82 | 47 | Control | n/a | IHC |
| 20 | M | 82 | 24 | Control | n/a | IHC |
| 21 | F | 81 | 30 | Control | n/a | WB/IHC |
| 22 | F | 73 | 27 | Control | n/a | WB/IHC |
| 23 | M | 83 | 31 | Control | n/a | WB/IHC |
| 24 | F | 81 | 42 | Control | n/a | WB/IHC |
| 25 | F | 79 | 56 | Control | n/a | IHC |
| 26 |  |  |  | Control | n/a | IHC |
| 27 | F | 51 |  | Control | *n/a* | WB/IHC |
