# Supplementary material for: Aberrant NSUN1 activity connects m5C-RNA modification to TDP-43 neurotoxicity in ALS/FTD
Source: Life Sci Alliance. 2025 Nov 4;9(1):e202503297. doi: 10.26508/lsa.202503297 (PMC12588883; doi:10.26508/lsa.202503297)
Supplement: Supplementary file 4 [file LSA-2025-03297_TableS3.docx]

**Aberrant NSUN1 Activity Connects m5C RNA Modification to TDP-43 Neurotoxicity in ALS/FTD**

Melissa Parra Torres^1^, Kumara Dissanayake^1^, James Gray^1^, Alistair J. Langlands^2^, Ridvan Kucuk^1^, Marek Gierlinski^3^, Claire Troakes^4,5^, Andrew King^5^, and Leeanne McGurk^1*^

^1^Molecular, Cell and Developmental Biology, School of Life Sciences, University of Dundee, Dow Street, Dundee DD1 5EH, UK

### ^2^National Phenotypic Screening Centre, School of Life Sciences, University of Dundee, Dow Street, Dundee DD1 5EH, UK

^3^Data Analysis Group, Division of Computational Biology, School of Life Sciences, University of Dundee, Dundee, DD1 5EH, UK

^4^Department of Basic and Clinical Neuroscience, Wohl Clinical Neuroscience Institute, Institute of Psychiatry, Psychology and Neuroscience, King’s College London, London SE5 9RX, UK

^5^London Neurodegenerative Diseases Brain Bank, SGDP Centre, PO65, Institute of Psychiatry, Psychology and Neuroscience, King’s College London, London SE5 8AF, UK

# ^6^King's College Hospital NHS Foundation Trust, Academic Neuroscience Centre

* To whom correspondence should be addressed: Dr Leeanne McGurk, Cell and Developmental Biology, School of Life Sciences, University of Dundee, Dow Street, Dundee DD1 5EH, UK. Email: [LMcgurk001@dundee.ac.uk](mailto:LMcgurk001@dundee.ac.uk)

**Supplemental Tables: Table S2-S5**

**Table S3**: TDP-43 pathology and assessment of NSUN1 in spinal cord tissue

| **Case #** | **Motor neuron TDP-43 pathology** | **Non-neuronal TDP-43 pathology** | **Motor neuron loss** | **NSUN1 in cytoplasmic aggregates** |
| --- | --- | --- | --- | --- |
| 2 | 1 | 2 | 2 | n |
| 3 | 0 | 1 | 3 | n |
| 4 | 2 | 1 | 3 | n |
| 5 | 0 | 1 | 3 | n |
| 6 | 2 | 1 | 3 | n |
| 7 | 1 | 1 | 2 | n |
| 8 | 0 | 1 | 2 | n |
| 9 | 2 | 2 | 1 | n |
| 10 | 2 | 1 | 1 | n |
| 11 | 3 | 1 | 3 | n |

Scale

TDP-43 pathology: 0 = no TDP-43 aggregates; 1= mild TDP-43 aggregates; 2= moderate TDP-43 pathology; 3=severe TDP-43 pathology

Motor neuron loss: 1= mild motor neuron loss; 2=moderate motor neurons loss; 3= severe motor neuron loss.
