# Supplementary material for: Aberrant NSUN1 activity connects m5C-RNA modification to TDP-43 neurotoxicity in ALS/FTD
Source: Life Sci Alliance. 2025 Nov 4;9(1):e202503297. doi: 10.26508/lsa.202503297 (PMC12588883; doi:10.26508/lsa.202503297)
Supplement: Supplementary file 5 [file LSA-2025-03297_TableS4.docx]

**Aberrant NSUN1 Activity Connects m5C RNA Modification to TDP-43 Neurotoxicity in ALS/FTD**

Melissa Parra Torres^1^, Kumara Dissanayake^1^, James Gray^1^, Alistair J. Langlands^2^, Ridvan Kucuk^1^, Marek Gierlinski^3^, Claire Troakes^4,5^, Andrew King^5^, and Leeanne McGurk^1*^

^1^Molecular, Cell and Developmental Biology, School of Life Sciences, University of Dundee, Dow Street, Dundee DD1 5EH, UK

### ^2^National Phenotypic Screening Centre, School of Life Sciences, University of Dundee, Dow Street, Dundee DD1 5EH, UK

^3^Data Analysis Group, Division of Computational Biology, School of Life Sciences, University of Dundee, Dundee, DD1 5EH, UK

^4^Department of Basic and Clinical Neuroscience, Wohl Clinical Neuroscience Institute, Institute of Psychiatry, Psychology and Neuroscience, King’s College London, London SE5 9RX, UK

^5^London Neurodegenerative Diseases Brain Bank, SGDP Centre, PO65, Institute of Psychiatry, Psychology and Neuroscience, King’s College London, London SE5 8AF, UK

# ^6^King's College Hospital NHS Foundation Trust, Academic Neuroscience Centre

* To whom correspondence should be addressed: Dr Leeanne McGurk, Cell and Developmental Biology, School of Life Sciences, University of Dundee, Dow Street, Dundee DD1 5EH, UK. Email: [LMcgurk001@dundee.ac.uk](mailto:LMcgurk001@dundee.ac.uk)

**Table S4**: Drosophila strains

| Label | Genotype | Source |
| --- | --- | --- |
| si.mCherry | y[1] sc[*] v[1] sev[21]; P{y[+t7.7] v[+t1.8]=VALIUM20-mCherry}attP2 | Bloomington, #35785 |
| gmr-GAL4 (YH3) | *w[*]; P{w[+mC]=GMR-GAL4.Y}YH3/TM3, Sb[1]* | Bloomington #84247 |
| gmr-GAL4 (II) | *w[*]; P{w[+mC]=GAL4-ninaE.GMR}12* | Bloomington 1104 |
| TDP-43 | *UAS-TDP-43 (37M)/CyO; gmr-GAL4(YH3)/TM6B* | Elden et al 2010, Kim et al 2014 |
| si.*Nsun*1 | *y[1] sc[*] v[1] sev[21]; P{y[+t7.7] v[+t1.8]=TRiP.HMC04440}attP40* | Bloomington # 56998 |
| si.*Nsun*2 | *y[1] v[1]; P{y[+t7.7] v[+t1.8]=TRiP.HMJ24019}attP40/CyO* | Bloomington # 62495 |
| si.*Nsun*4 | *n[1] P{ry[+t7.2]=PZ }l(2)10685[10685]/CyO; ry[506]* | Bloomington # 12381 |
| si.*Nsun*5 | *y[1] sc[*] v[1] sev[21]; P{y[+t7.7] v[+t1.8]=TRiP.HMS00438}attP2/TM3, Sb[1]* | Bloomington # 32440 |
| si*.Nsun6* | *y[1] sc[*] v[1] sev[21]; P{y[+t7.7] v[+t1.8]=TRiP.HMC04118}attP40* | Bloomington # 56897 |
| si.*Mt2* | \|  \| y[1] sc[*] v[1] sev[21]; P{y[+t7.7] v[+t1.8]=TRiP.HMS02599}attP40 \| \| --- \| --- \| | Bloomington # 42906 |
| LacZ | *w-; UAS-LacZ; gmr-GAL4 (YH3)* | Elden et al 2010, Kim et al 2014 |
| DaGS | *w-;Daugterless-GAL4-geneswitch; +/+* | (Tricoire *et al*, 2009) |
| Da-Gal4 | w*; Daughterless-GAL4/TM6c, Sb1, | Bloomington # 55851 |
| D42-GAL4 | \|  \| w[*]; P{w[+mW.hs]=GawB}D42 \| \| --- \| --- \| | Bloomington #8816 |
| elav3A-GAL4 | w[*];+/+;elav3A-GAL4/Sb | (Hekmat-Scafe *et al*, 2005) |
| repo-GAL4 | w[1118]; P{w[+mC]=repo-Gal4.L}Oatp30B[repoF3] | Bloomington # 602908 |
| 24B –GAL4 | w[*]; P{w[+mW.hs]=GawB}how[24B] | Bloomington # 1767 |
| 5X-TDP-43 | w[*]; 5X-UAS-TDP-43^attP40^/+ | This study |
| UAS-CAG_76_ | w+;UAS-SCA3trQ78 (c11.2);rh1-Gal4/TM6,Tb | (Warrick *et al*, 1998) |
| UAS-ATXN1-CAG_82_ | w[1118]; P{w[+mC]=UAS-Hsap\ATX1.82Q}M6 | Bloomington # 33818 |
| UAS-(G4C2)_48_ | w[1118]; ; UAS-(G4C2)48#3.1 (III)/TM6c,sb1 | (Goodman *et al*, 2019) |
|  |  |  |
