# Supplementary material for: Aberrant NSUN1 activity connects m5C-RNA modification to TDP-43 neurotoxicity in ALS/FTD
Source: Life Sci Alliance. 2025 Nov 4;9(1):e202503297. doi: 10.26508/lsa.202503297 (PMC12588883; doi:10.26508/lsa.202503297)
Supplement: Supplementary file 6 [file LSA-2025-03297_TableS5.docx]

**Aberrant NSUN1 Activity Connects m5C RNA Modification to TDP-43 Neurotoxicity in ALS/FTD**

Melissa Parra Torres^1^, Kumara Dissanayake^1^, James Gray^1^, Alistair J. Langlands^2^, Ridvan Kucuk^1^, Marek Gierlinski^3^, Claire Troakes^4,5^, Andrew King^5^, and Leeanne McGurk^1*^

^1^Molecular, Cell and Developmental Biology, School of Life Sciences, University of Dundee, Dow Street, Dundee DD1 5EH, UK

### ^2^National Phenotypic Screening Centre, School of Life Sciences, University of Dundee, Dow Street, Dundee DD1 5EH, UK

^3^Data Analysis Group, Division of Computational Biology, School of Life Sciences, University of Dundee, Dundee, DD1 5EH, UK

^4^Department of Basic and Clinical Neuroscience, Wohl Clinical Neuroscience Institute, Institute of Psychiatry, Psychology and Neuroscience, King’s College London, London SE5 9RX, UK

^5^London Neurodegenerative Diseases Brain Bank, SGDP Centre, PO65, Institute of Psychiatry, Psychology and Neuroscience, King’s College London, London SE5 8AF, UK

# ^6^King's College Hospital NHS Foundation Trust, Academic Neuroscience Centre

* To whom correspondence should be addressed: Dr Leeanne McGurk, Cell and Developmental Biology, School of Life Sciences, University of Dundee, Dow Street, Dundee DD1 5EH, UK. Email: [LMcgurk001@dundee.ac.uk](mailto:LMcgurk001@dundee.ac.uk)

**Table S5**: Drosophila genotypes related to Figures 2,3,5 and S6.

| Figure | Label | Genotype |
| --- | --- | --- |
| 2D | control | *y, sc, v, sev/w^1118^; Daughterless-GAL4-geneswitch/+; si.mCherry^35785^/+* |
|  | si.*Nsun1* | *y, sc, v, sev/w^1118^; Daughterless-GAL4-geneswitch/)/ si.NSsun1^TRiP.HMC04440^; +/+* |
| 2E | control | *w^1118^; +/+; Daughterless-GAL4/si.mCherry^35785^* |
|  | si.*Nsun2* | *w^1118^; si.Nsun2 ^TRiP.HMJ24019^/+; Daughterless-GAL4 /+* |
|  | si.*Nsun4* | *w^1118^; cn[1] l(2)10685[10685]/+; Daughterless-GAL4 /+* |
|  | si.*Nsun5* | *w^1118^; +/+; Daughterless-GAL4/si.Nsun5^TRiP.HMS00438^* |
|  | si.*Nsun6* | *w^1118^; +/+; Daughterless-GAL4/si.Nsun6^TRiP.HMC04118^* |
|  | si.*Mt2* | *w^1118^; si.mt2^TRiP.^ ^HMS0166^/+; Daughterless-GAL4/+* |
| 3 A, B, D | gmr>control | *y, sc, v, sev/w^1118^; +/+; gmr-GAL4 (YH3)/si.mCherry^35785^* |
|  | ctrl | *y, sc, v, sev/w^1118^; UAS-TDP-43/+; gmr-GAL4 (YH3)/ si.mCherry^35785^* |
|  | si.*Nsun1* | *y, sc, v, sev/w^1118^; UAS-TDP-43 (37M)/ si.Nsun1^TRiP.HMC04440^; gmr-GAL4 (YH3)/+,* |
|  | si.*Nsun2* | *y, sc, v, sev/w^1118^; TDP-43 (37M)/ si.Nsun1^TRiP.HMJ24019^; gmr-GAL4 (YH3)/+,* |
|  | si.*Nsun4* | *y, sc, v, sev/w^1118^; TDP-43 (37M)/cn[1] l(2)10685[10685]; gmr-GAL4 (YH3)/+,* |
|  | si.*Nsun5* | *y, sc, v, sev/w^1118^; TDP-43 (37M)/+; gmr-GAL4 (YH3)/si.Nsun5^TRiP.HMS00438^,* |
|  | si.*Nsun6* | *y, sc, v, sev/w^1118^; TDP-43 (37M)/ si.Nsun6^TRiP.HMC04118^; gmr-GAL4 (YH3)/+,* |
|  | si.*Mt2* | *y, sc, v, sev/w^1118^; si.Mt2: TDP-43 (37M)/ si.Mt2^TRiP.^ ^HMS01667^_,_* |
| 4A-E | elavGS > control | *w*; +/+; elavGS/si.mCherry^35785^* |
|  | elavGS > si.*Nsun1* | *w*; +/ si.Nsun1^TRiP.HMJ24019^; elavGS/+.* |
|  | elavGS >TDP-43 + si.Nsun1 | *w*; +/ si.Nsun1^TRiP.HMJ24019^; elavGS, UAS-TDP-43-52S/+.* |
|  | elavGS >TDP-43 + control | *w*; +/+; elavGS, UAS-TDP-43-52S/si.mCherry^35785^* |
| 4G-I | elavGS > control | *w*; +/+; elavGS/si.mCherry^35785^* |
|  | elavGS > TDP-43 + control: | *w*; UAS-TDP-43-5X /+; elavGS/si.mCherry^35785^* |
|  | elavGS > 5X-TDP-43 + si.*Nsun1* | : *w*; UAS-TDP-43-5X/si.Nsun1^TRiP.HMJ24019^; elavGS/+.* |
| 5B-F | gmr>control | *y, sc, v, sev/w^1118^; +/+; gmr-GAL4 (YH3)/si.mCherry^35785^* |
|  | gmr> TDP-43 + control | *y, sc, v, sev/w^1118^; UAS-TDP-43* (37M)*/ +; gmr-GAL4 (YH3)/ si.mCherry^35785^* |
|  | gmr> TDP-43 + si. *Nsun1* | *y, sc, v, sev/w^1118^; UAS-TDP-43* (37M)*/ si.Nsun1^TRiP.HMC04440^; gmr-GAL4 (YH3)/+,* |
| S6B | control | *w-;+/+; daGS-GAL4/si.mCherry^35785^* |
|  | si.*Nsun1* | *w-; si.Nsun1*^TRiP.HMC04440^/+; *daGS-GAL4/+* |
| S6C-E | gmr>control | *y, sc, v, sev/w^1118^; +/+; gmr-GAL4 (YH3)/si.mCherry^35785^* |
|  | gmr> TDP-43 Q331K + control | *y, sc, v, sev/w^1118^; UAS-TDP-43* Q331K*/ +; gmr-GAL4 (YH3)/ si.mCherry^35785^* |
|  | gmr> TDP-43 Q331K + si.*Nsun1* | *y, sc, v, sev/w^1118^; UAS-TDP-43* Q331K*/ si.NSUN1^TRiP.HMC04440^; gmr-GAL4 (YH3)/+,* |
| S6D | control | *w-;+/+; daGS-GAL4/si.mCherry^35785^* |
|  | si.*Nsun1* | *w-; si.Nsun1*^TRiP.HMC04440^/+; *daGS-GAL4/+* |
| S6F-G | gmr>control | *y, sc, v, sev/w^1118^; +/+; gmr-GAL4 (YH3)/si.mCherry^35785^* |
|  | gmr> TDP-43 + control | *y, sc, v, sev/w^1118^; UAS-TDP-43/+; gmr-GAL4 (YH3)/ si.mCherry^35785^* |
|  | gmr> TDP-43 + si. *Nsun1* | *y, sc, v, sev/w^1118^; UAS-TDP-43 (37M)/ si.NSUN1^TRiP.HMC04440^; gmr-GAL4 (YH3)/+,* |
| S6H-I | gmr> lacZ + control | *w*; gmr-GAL4, UAS-LacZ)/+; +/ si.mCherry^35785^* |
|  | gmr> lacZ + si.*Nsun1* | *w*; gmr-GAL4, UAS-LacZ)/ si.Nsun1^TRiP.HMC04440^; +/+.* |
| S6J | gmr-GAL4 > control | *y, sc, v, sev/w^1118^; +/+; gmr-GAL4 (YH3)/si.mCherry^35785^* |
|  | gmr-GAL4 > si.Nsun1 | *y, sc, v, sev/w^1118^; +/ si.Nsun1^TRiP.HMC04440^; gmr-GAL4 (YH3)/+* |
|  | gmr-GAL4 > UAS-CAG78 + control | *y, sc, v, sev/w^1118^; )/+; gmr-GAL4 (YH3),*UAS-CAG78/ *si.mCherry^35785^* |
|  | gmr-GAL4 > UAS-CAG78 + si.Nsun1 | *y, sc, v, sev/w^1118^; si.Nsun1^TRiP.HMC04440^/+; gmr-GAL4 (YH3),*UAS-CAG78/+ |
|  | gmr-GAL4 > UAS-ATXN1-CAG82 + control | *y, sc, v, sev/w^1118^; +/+; gmr-GAL4 (YH3),* UAS-ATXN1-CAG82/ *si.mCherry^35785^* |
|  | gmr-GAL4 > UAS-ATXN1-CAG82 + si.Nsun1 | *y, sc, v, sev/w^1118^; si.Nsun1^TRiP.HMC04440^/+; gmr-GAL4 (YH3),* UAS-ATXN1-CAG82/+ |
|  | gmr-GAL4 > UAS-(G4C2)_48_ + control | *y, sc, v, sev/w^1118^; +/+; gmr-GAL4 (YH3),* UAS-(G4C2)_48_/ *si.mCherry^35785^* |
|  | gmr-GAL4 > UAS-(G4C2)_48_ + si.Nsun1 | *y, sc, v, sev/w^1118^; si.Nsun1^TRiP.HMC04440^/+; gmr-GAL4 (YH3),* UAS-(G4C2)_48_/+ |
| S6K | da-GAL4 > control | *y, sc, v, sev/w^1118^; +/+; Daughterless-GAL4/si.mCherry^35785^* |
|  | da-GAL4 > si.Nsun1 | *y, sc, v, sev/w^1118^; si.Nsun1*^TRiP.HMC04440^*/+; Daughterless-GAL4/+* |
|  | elav3A > control | *y, sc, v, sev/w^1118^; +/+; elav3A-GAL4/ si.mCherry^35785^* |
|  | elav3A > si.Nsun1 | *y, sc, v, sev/w^1118^; si.Nsun1*^TRiP.HMC04440^*/+; elav3A-GAL4/+* |
|  | D42-GAL4 > control | *y, sc, v, sev/w^1118^; +/+;D42-GAL4/si.mCherry^35785^* |
|  | D42-GAL4 > si.Nsun1 | *y, sc, v, sev/w^1118^; si.Nsun1*^TRiP.HMC04440^*/+; D42-GAL4/+* |
|  | repo-GAL4 > control | *y, sc, v, sev/w^1118^; +/+;repo-GAL4/si.mCherry^35785^* |
|  | repo-GAL4 > si.Nsun1 | *y, sc, v, sev/w^1118^; si.Nsun1*^TRiP.HMC04440^*/+; repo-GAL4/+* |
|  | 24B-GAL4 > control | *y, sc, v, sev/w^1118^; +/+;*24B*-GAL4/si.mCherry^35785^* |
|  | 24B-GAL4> si.Nsun1 | *y, sc, v, sev/w^1118^; si.Nsun1*^TRiP.HMC04440^*/+;* 24B*-GAL4/+* |
|  | gmr-GAL4 > control | *y, sc, v, sev/w^1118^; +/+; gmr-GAL4 (YH3)/si.mCherry^35785^* |
|  | gmr-GAL4 > si.Nsun1 | *y, sc, v, sev/w^1118^; si.Nsun1^TRiP.HMC04440^/+; gmr-GAL4 (YH3)/+* |
