# Supplementary material for: Aberrant NSUN1 activity connects m5C-RNA modification to TDP-43 neurotoxicity in ALS/FTD
Source: Life Sci Alliance. 2025 Nov 4;9(1):e202503297. doi: 10.26508/lsa.202503297 (PMC12588883; doi:10.26508/lsa.202503297)
Supplement: Supplementary file 7 [file LSA-2025-03297_TableS6.docx]

**Aberrant NSUN1 Activity Connects m5C RNA Modification to TDP-43 Neurotoxicity in ALS/FTD**

Melissa Parra Torres^1^, Kumara Dissanayake^1^, James Gray^1^, Alistair J. Langlands^2^, Ridvan Kucuk^1^, Marek Gierlinski^3^, Claire Troakes^4,5^, Andrew King^5^, and Leeanne McGurk^1*^

^1^Molecular, Cell and Developmental Biology, School of Life Sciences, University of Dundee, Dow Street, Dundee DD1 5EH, UK

### ^2^National Phenotypic Screening Centre, School of Life Sciences, University of Dundee, Dow Street, Dundee DD1 5EH, UK

^3^Data Analysis Group, Division of Computational Biology, School of Life Sciences, University of Dundee, Dundee, DD1 5EH, UK

^4^Department of Basic and Clinical Neuroscience, Wohl Clinical Neuroscience Institute, Institute of Psychiatry, Psychology and Neuroscience, King’s College London, London SE5 9RX, UK

^5^London Neurodegenerative Diseases Brain Bank, SGDP Centre, PO65, Institute of Psychiatry, Psychology and Neuroscience, King’s College London, London SE5 8AF, UK

# ^6^King's College Hospital NHS Foundation Trust, Academic Neuroscience Centre

* To whom correspondence should be addressed: Dr Leeanne McGurk, Cell and Developmental Biology, School of Life Sciences, University of Dundee, Dow Street, Dundee DD1 5EH, UK. Email: [LMcgurk001@dundee.ac.uk](mailto:LMcgurk001@dundee.ac.uk)

**Supplemental Tables: Table S2-S5**

**Table S6: Primers**

| QuikChange primers | |
| --- | --- |
| TDP-43-Q331K-FP | 5’GCCCAGGCAGCACTAAAGAGCAGTTGGGG3’ |
| TDP-43-Q331K-RP | 5’CCCCAACTGCTCTTTAGTGCTGCCTGGGC3’ |
| Real time PCR | |
| Nop2/NSUN1 FP1 | 5’ ATGGCACAAACTCCCCTAGC 3’ |
| Nop2/NSUN1 RP1 | 5’ CAGTTTGGCGTTTCCCTTGG 3’ |
| Q28S-F | 5' GTCAGGGGAAACCCTGATGG 3’ |
| Q28S-R | 5' ATCGTTTCGACCCTAAGGCC 3' |
| Q5.8S-F | 5’ CTAGGCGGTGGATCACTCGG 3' |
| Q5.8S-R | 5' CAGCATGGACTGCGATATGC 3’ |
| Q18S-F | 5' GGCTAAAACCAAGCGATCGC 3' |
| Q18S-R | 5' CTCCCTCTCCGGAATCGAAC 3' |
| QITS1-F | 5' TTATTGAAGGAATTGATATATGCC 3’ |
| QITS1-R | 5' ATGAGCCGAGTGATCCAC 3’ |
| QETS-F | 5' GCTCCGCGGATAATAGGAAT 3' |
| QETS-R | 5' ATATTTGCCTGCCACCAAAA 3' |
| α-Tubulin F | 5’ CATCCAAGCTGGTCAGTG 3' |
| α-Tubulin R | 5’ GCCATGCTCATCGGAGAT 3’ |
| 25S rRNA PCR FP2 | 5’ GGT GTT TAG TAT ATA TAG GGA TAG TAA A 3’ |
| 25S rRNA PCR RP2 | 5’ TTC CCT TAC ATA AAT AAA CAA TCC AAC 3’ |
| NSUN2 FP1 | 5’CACACTGCGCAAGAATCCAG 3’ |
| NSUN2 RP1 | 5’AATGCCGTGGAGGTTGTAGG 3’ |
| NSUN4 FP1 | 5’TTCACGGAGTACGGCATCAC 3’ |
| NSUN4 RP1 | 5’TAGGGCACCACCATTTGTCC 3’ |
| NSUN5 FP1 | 5’GAGACGCCTTGAACCtAACAC 3’ |
| NSUN5 RP1 | 5’ACACGGTCATGCGGTTTTG 3’ |
| NSUN6 FP1 | 5’AGTCAAACACTGAGCGGGAG 3’ |
| NSUN6 RP1 | 5’AACACCTTGCCTGTACCCAG 3’ |
| MT2 FP2 | 5’TTGCAAAGGGACACGGAAGA3’ |
| MT2 RP2 | 5’AGCTCTCGAAACCCTTGACG 3’ |
| Dgt5 FP2 | 5’CCCGAAAAGAGTACCAAAAGCT 3’ |
| Dgt5 RP2 | 5’GGCTGGGATATTGCATTTGGAT 3’ |
