# Supplementary material for: Aberrant NSUN1 activity connects m5C-RNA modification to TDP-43 neurotoxicity in ALS/FTD
Source: Life Sci Alliance. 2025 Nov 4;9(1):e202503297. doi: 10.26508/lsa.202503297 (PMC12588883; doi:10.26508/lsa.202503297)
Supplement: Supplementary file 1 [file LSA-2025-03297_SdataF1_F4_F5_F6_F7_F8_FS9.docx]

**Aberrant NSUN1 Activity Connects m5C RNA Modification to TDP-43 Neurotoxicity in ALS/FTD**

Melissa Parra Torres^1^, Kumara Dissanayake^1^, James Gray^1^, Alistair J. Langlands^2^, Ridvan Kucuk^1^, Marek Gierlinski^3^, Claire Troakes^4,5^, Andrew King^5^, and Leeanne McGurk^1*^

^1^Molecular, Cell and Developmental Biology, School of Life Sciences, University of Dundee, Dow Street, Dundee DD1 5EH, UK

### ^2^National Phenotypic Screening Centre, School of Life Sciences, University of Dundee, Dow Street, Dundee DD1 5EH, UK

^3^Data Analysis Group, Division of Computational Biology, School of Life Sciences, University of Dundee, Dundee, DD1 5EH, UK

^4^Department of Basic and Clinical Neuroscience, Wohl Clinical Neuroscience Institute, Institute of Psychiatry, Psychology and Neuroscience, King’s College London, London SE5 9RX, UK

^5^London Neurodegenerative Diseases Brain Bank, SGDP Centre, PO65, Institute of Psychiatry, Psychology and Neuroscience, King’s College London, London SE5 8AF, UK

# ^6^King's College Hospital NHS Foundation Trust, Academic Neuroscience Centre

* To whom correspondence should be addressed: Dr Leeanne McGurk, Cell and Developmental Biology, School of Life Sciences, University of Dundee, Dow Street, Dundee DD1 5EH, UK. Email: [LMcgurk001@dundee.ac.uk](mailto:LMcgurk001@dundee.ac.uk)

**Raw panel data**

­­­
